# Cluster Analysis of Cell Nuclei in H&E-Stained Histological Sections of Prostate Cancer and Classification Based on Traditional and Modern Artificial Intelligence Techniques

**DOI:** 10.3390/diagnostics12010015

**Published:** 2021-12-22

**Authors:** Subrata Bhattacharjee, Kobiljon Ikromjanov, Kouayep Sonia Carole, Nuwan Madusanka, Nam-Hoon Cho, Yeong-Byn Hwang, Rashadul Islam Sumon, Hee-Cheol Kim, Heung-Kook Choi

**Affiliations:** 1 Department of Computer Engineering, u-AHRC, Inje University, Gimhae 50834, Korea; subrata_bhattacharjee@outlook.com; 2 Department of Digital Anti-Aging Healthcare, u-AHRC, Inje University, Gimhae 50834, Korea; kobiljonikromjanov@gmail.com (K.I.); carolesonia39@gmail.com (K.S.C.); hyb1345679@gmail.com (Y.-B.H.); sumon39.cst@gmail.com (R.I.S.); heeki@inje.ac.kr (H.-C.K.); 3 School of Computing & IT, Sri Lanka Technological Campus, Paduka 10500, Sri Lanka; nuwanmadusanka@hotmail.com; 4 Department of Pathology, Yonsei University Hospital, Seoul 03722, Korea; cho1988@yumc.yonsei.ac.kr

**Keywords:** histopathology, prostate cancer, segmentation, cluster analysis, artificial intelligence, classification

## Abstract

Biomarker identification is very important to differentiate the grade groups in the histopathological sections of prostate cancer (PCa). Assessing the cluster of cell nuclei is essential for pathological investigation. In this study, we present a computer-based method for cluster analyses of cell nuclei and performed traditional (i.e., unsupervised method) and modern (i.e., supervised method) artificial intelligence (AI) techniques for distinguishing the grade groups of PCa. Two datasets on PCa were collected to carry out this research. Histopathology samples were obtained from whole slides stained with hematoxylin and eosin (H&E). In this research, state-of-the-art approaches were proposed for color normalization, cell nuclei segmentation, feature selection, and classification. A traditional minimum spanning tree (MST) algorithm was employed to identify the clusters and better capture the proliferation and community structure of cell nuclei. K-medoids clustering and stacked ensemble machine learning (ML) approaches were used to perform traditional and modern AI-based classification. The binary and multiclass classification was derived to compare the model quality and results between the grades of PCa. Furthermore, a comparative analysis was carried out between traditional and modern AI techniques using different performance metrics (i.e., statistical parameters). Cluster features of the cell nuclei can be useful information for cancer grading. However, further validation of cluster analysis is required to accomplish astounding classification results.

## 1. Introduction

Many techniques are used for analysis, color enhancement, segmentation, and classification of medical images, such as those yielded by magnetic resonance (MR), positron emission tomography (PET), and microscopic biopsy; many internal bodily structures can be imaged non-invasively. Computers can be used for image gain, storage, presentation, and communication. Clinical, biochemical, and pathological images are used to diagnose and stage PCa; computer scientists are very active in this field. However, the sensitivity and specificity of the techniques remain controversial [1]. PCa diagnosis requires prostate MR and microscopic biopsy images. A traditional cancer diagnosis is subjective; pathologists examine biopsy samples under a microscope. It is difficult to objectively describe tissue texture, tissue color, and cell morphology.

Despite recent advances, PCa remains a major medical issue among males, being associated with the overtreatment of inherently benign disease and inadequate treatment of metastases [2]. The prostate has a pseudostratified epithelium with three types of terminally differentiated epithelial cells: luminal, basal, and neuroendocrine [3]. Other cells of the epithelium include fibroblasts, smooth muscle cells, endothelial cells, immune cells, autonomic nerve fibers, and associated ganglia [4]. Malignant transformation is a multistage process; prostatic intraepithelial neoplasia (PIN) triggers localized PCa followed by adenocarcinoma characterized by local invasion and, finally, metastatic PCa. The most common PCa grading system is the Gleason system, which has been refined since it was first introduced in 1974 [5]; the system is widely used to score PCa aggressiveness. However, there are problems, including inter- and intra-observer variation. In addition, most biopsy samples are negative [6,7,8]. Here, we evaluate histopathological images of cancerous tissues. PCa grading was performed by a pathologist based on structural changes in stained sections.

Computer-based algorithms can perform cluster analyses of cell nuclei; available methods include traditional MST [9,10,11]. MST cluster analysis, derived from graph theory, explores nuclear distributions. A tree is used to represent binary relationships; the connected components constitute a subtree representing an independent cluster. The identification of cancer cell abnormalities is essential for early cancer detection. Today, ML and deep learning (DL) algorithms are used for medical image analysis, feature classification, and pattern recognition. ML algorithms are usually accurate, fast, and customizable. ML iteration is essential; new data must be received and assimilated. Supervised learning is commonly used during ML training and testing; a model is trained using labeled data in a training set, and the knowledge thus acquired is used to evaluate unforeseen labeled data in a test set [12]. On the other hand, unsupervised learning is not commonly used for the prediction of the diagnosis of different diseases. It is essential in the real-world environment and discovers hidden patterns using the unlabeled datasets. Therefore, unsupervised learning is also a trustworthy method but computationally complex.

In this study, four state-of-the-art approaches were proposed for color normalization, cell nuclei segmentation, feature selection, and ML classification. Histopathology samples were collected from two different centers and created two datasets for binary (grade 3 vs. grade 5) and multiclass (grade 3 vs. grade 4 vs. grade 5) classification. Before we perform the segmentation, stain normalization and deconvolution techniques were carried out as a preprocessing step. After stain deconvolution, the image hematoxylin channel was selected for extracting the cell nuclei tissue components. Furthermore, we used an advanced method (i.e., marker-controlled watershed algorithm) to separate the overlapping cell nuclei. Next, we use an MST algorithm to perform cluster analysis and extract significant information for AI classification. The cell nuclei clusters were separated, and their features are evaluated heuristically. Cluster analysis was performed to better capture the proliferation and community structure of cell nuclei. These methods are making their way into pathology via various computer-aided detection (CAD) systems to assist pathologic diagnosis. Then, we proposed a majority voting method by combining filter and wrapper-based techniques for selecting the most significant features. Finally, we use state-of-the-art algorithms (i.e., stacked ML ensemble and k-medoids clustering) to perform supervised and unsupervised PCa classification. The performance metrics used for evaluating the results are accuracy, precision, recall, and F1-score.

The remainder of this paper are as follows: Section 2 presents the related work of the past study where we discussed different state-of-the-art methods for PCa analysis. Section 3 illustrates the materials and methods of the study where we mentioned the process of data collection and state-of-the-art techniques used in this study. In Section 4, we presented the results of AI models and discussed the overall implication of the study. Lastly, the paper is concluded in Section 5.

## 2. Related Work

Histopathology image analysis of PCa is quite problematic compared to other cancer types. Many researchers are still working on it and trying to develop new techniques for detecting and treating PCa. It is very difficult to analyze PCa under a microscope based on the Gleason grading system because the tissue pattern, formation of the gland, and distribution of cell nuclei is quite similar in some regions (i.e., score 3 and 4) of the whole slide image (WSI). Most of the existing research performed texture and morphological analysis to differentiate cancer scoring using histopathology images. Table 1 shows the summary of the significant papers that used microscopy biopsy tissue images for the analysis of PCa.

The studies in Table 1 confirm the success of the analysis of histopathological images for the classification of PCa such as benign vs. malignant and low- vs. high-grade cancer. It has been analyzed from the above-mentioned studies that most of the authors performed morphological and texture feature analysis for PCa classification. However, it has also been shown that morphological analysis of cell nuclei is not significant for PCa diagnosis because the shape and size of the cell nucleus are almost similar in all the grades (i.e., grade 3, grade 4, and grade 5), and AI models can produce unsatisfactory results. Therefore, in the present study, we performed the PCa analysis only based on the cluster features of the cell nuclei. The features extracted from the clusters are provided in Section 3.2.4.

## 3. Materials and Methods

### 3.1. Data Acquisition

Dataset 1 (grade 3, grade 4, and grade 5 WSIs) was collected from the Yonsei University Severance Hospital, Korea. WSIs were scanned into a computer at 40× optical magnification using a 0.3 NA objective, fitted to a C-3000 digital camera (Olympus, Tokyo, Japan) attached to a BX-51 microscope (Olympus). The tissue samples had been sectioned to a thickness of 4 μm; then, the sections were deparaffinized, rehydrated, and stained with H&E (staining blue and red, respectively). The WSIs used for this research were acquired from 80 patients.

Dataset 2 (grade 3, grade 4, and grade 5 WSIs) was collected from the Kaggle repository, available at https://www.kaggle.com/c/prostate-cancer-grade-assessment (accessed on 25 March 2021). The WSIs were analyzed and prepared at Radboud University medical center. All the slides were scanned using 3DHistech Panoramic Flash II 250 scanner at 20× magnification (pixel resolution 0.48 μm). All cases were retrieved from the pathology achieves of the Radboud University Medical Center. Patients with a pathologist’s report between 2012 and 2017 were eligible for inclusion. The WSIs used for this research were acquired from 60 patients.

**Figure 1:**
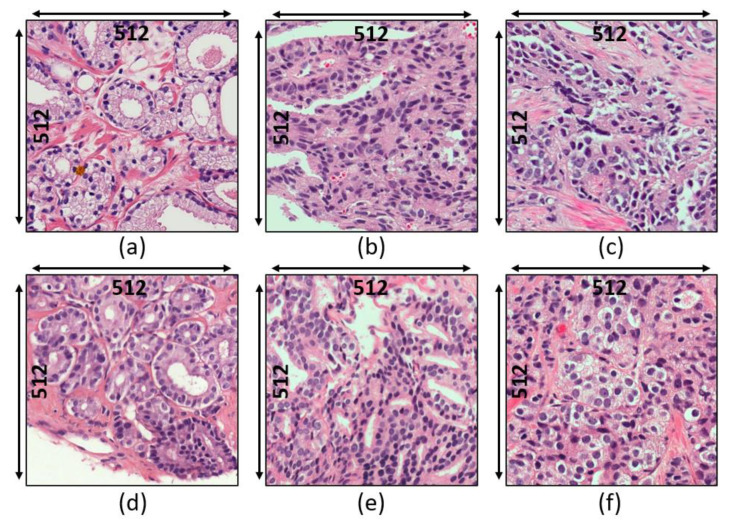
Histologic findings for each grade of prostate cancer. (**a**–**c**) Dataset 1: grade 3, grade 4, and grade 5, respectively. (**d**–**f**) Dataset 2: grade 3, grade 4, and grade 5, respectively.

**Figure A1:**
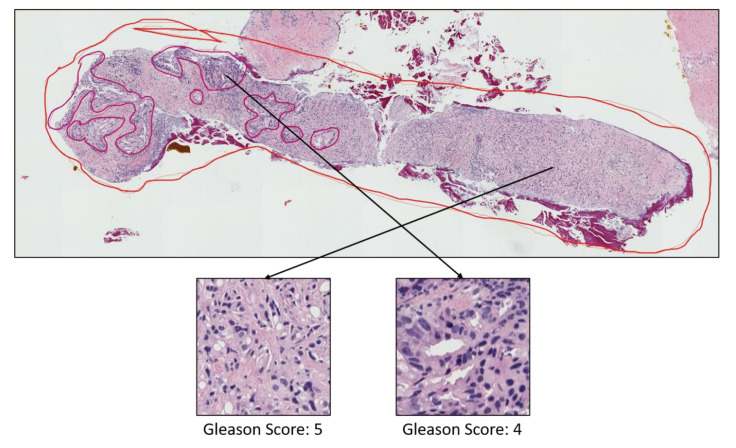
Prostate adenocarcinoma with Gleason scores 4 and 3 annotated with red and blue color, respectively.

**Figure A2:**
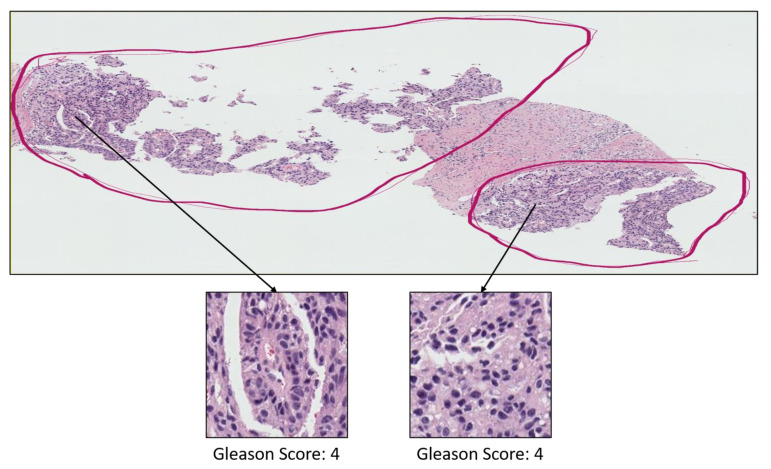
Prostate adenocarcinoma with Gleason scores 4 annotated with red color.

**Figure A3:**
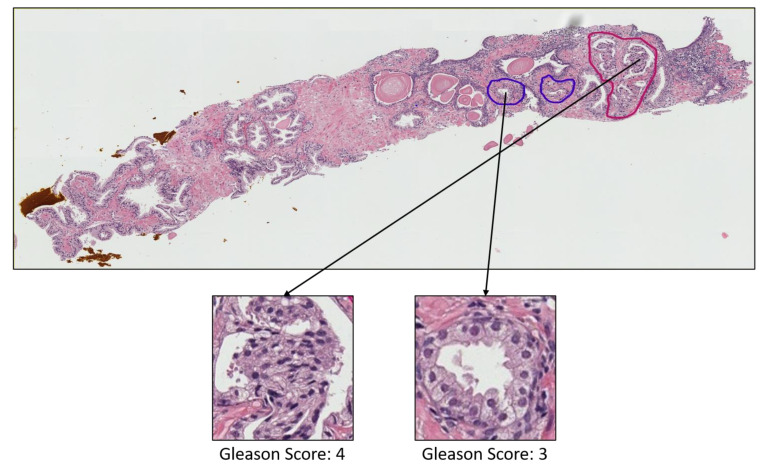
Prostate adenocarcinoma with Gleason scores 5 and 4 annotated with orange and red color, respectively.

A total of 900 H&E-stained patch images of size 512 × 512 pixels were generated by tiling the pathology annotated slides. Furthermore, the acquired samples were divided equally into three cancer grades (300 grade 3, 300 grade 4, and 300 grade 5). For supervised classification, the dataset was divided into two subsets: train set (80%) and test set (20%). On the other hand, unsupervised classification was performed using the whole dataset. Examples of histopathological images of datasets 1 and 2 are shown in Figure 1. The binary classification was defined (grade 3 vs. grade 5) as was multiclass classification (grade 3 vs. grade 4 vs. grade 5). Appendix A, Figure A1, Figure A2 and Figure A3 show the illustration of the Gleason grading process. Each of the grades is assigned according to the Gleason grading system as follows:

- Grade 3: Gleason score 4 + 3 = 7. Distinctly infiltrative margin.
- Grade 4: Gleason score 4 + 4 = 8. Irregular masses of neoplastic glands. Cancer cells have lost their ability to form glands.
- Grade 5: Gleason score 4 + 5, 5 + 4, or 5 + 5 = 9 or 10. Only occasional gland formation. Sheets of cancer cells throughout the tissue.

### 3.2. Research Pipeline

**Figure 2:**
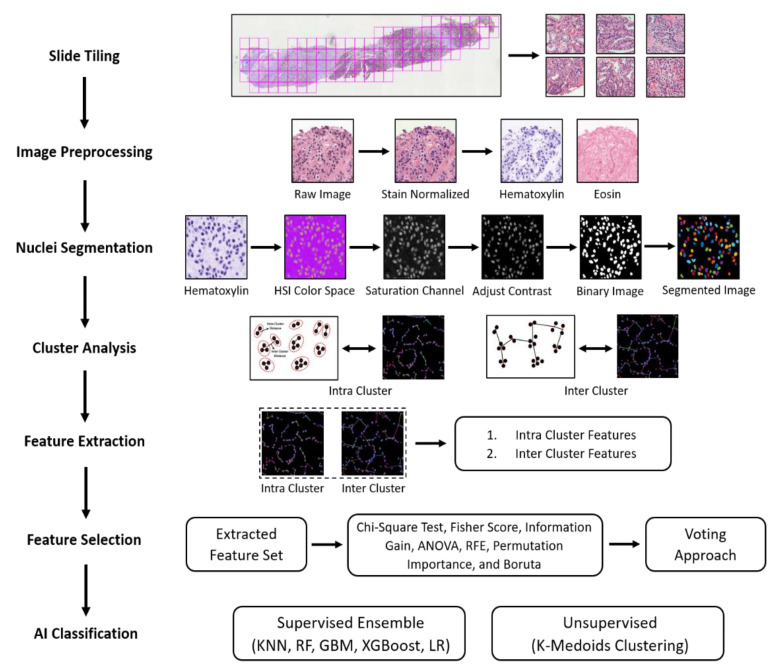
Analytical pipeline for the cluster analysis and AI classification of cancer grades observed in histological sections.

The patch images of size 512 × 512 pixels were extracted to perform AI classification. Figure 2 illustrates the entire methodology for AI classification to distinguish between the grades of PCa. The pipeline plotted below consisted of seven phases, which include slide tiling, image preprocessing, nuclei segmentation, cluster analysis, feature extraction, feature selection, and AI classification.

#### 3.2.1. Image Preprocessing

**Figure 3:**
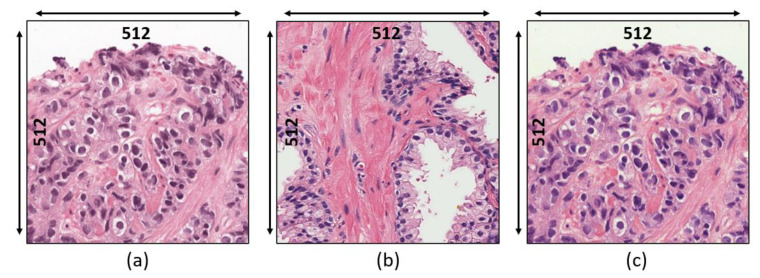
Stain normalization. (**a**) Raw image. (**b**) Reference image. (**c**) Normalized image.

Our observations on H&E-stained images show that there is a problem of color constancy, and it is a critical issue for segmentation. Therefore, stain normalization represents a vital step for balancing the color intensity in the histological section. We applied stain normalization and stain deconvolution techniques as a preprocessing step. To perform stain normalization, we selected an image from the dataset as a reference image to match the color intensity with the source images in the dataset. Therefore, the stain normalization approach was applied by transforming both the source and reference image to the LAB color space, and the mean and standard deviation of the reference image are harmonized to that of the source image. Figure 3 shows the source, reference, and normalized images. Based on the statistics of the source and reference images, each image channel was normalized. However, to improve the quality of the images, the computation process of stain normalization has been slightly modified from the original equations and can be expressed as:

(1) $$NormL_{map}=\left(\left(L_{src}-{\bar{L}}_{src}\right)\times \left(\frac{{\hat{L}}_{tar}}{{\hat{L}}_{src}}\right)\right)+\left(L_{src}+{\bar{L}}_{tar}\right)/2$$

(2) $$NormA_{map}=\left(\left(A_{src}-{\bar{A}}_{src}\right)\times \left(\frac{{\hat{A}}_{tar}}{{\hat{A}}_{src}}\right)\right)+\left(A_{src}+{\bar{A}}_{tar}\right)/2$$

(3) $$NormB_{map}=\left(\left(B_{src}-{\bar{B}}_{src}\right)\times \left(\frac{{\hat{B}}_{tar}}{{\hat{B}}_{src}}\right)\right)+\left(B_{src}+{\bar{B}}_{tar}\right)/2$$

(4) $$Norm_{map}=concateate\left(NormL_{map},NormA_{map},NormB_{map}\right)$$

where $\bar{L}$, $\bar{A}$, and $\bar{B}$ are the channel means and $\hat{L}$, $\hat{A}$, and $\hat{B}$ are the channel standard deviation, src is the source image, tar is the target image, and $Norm_{map}$ is the normalized LAB image, which was further converted to RGB color space. The end part of Equations (1)–(3) has been modified from the original equations [23].

**Figure 4:**
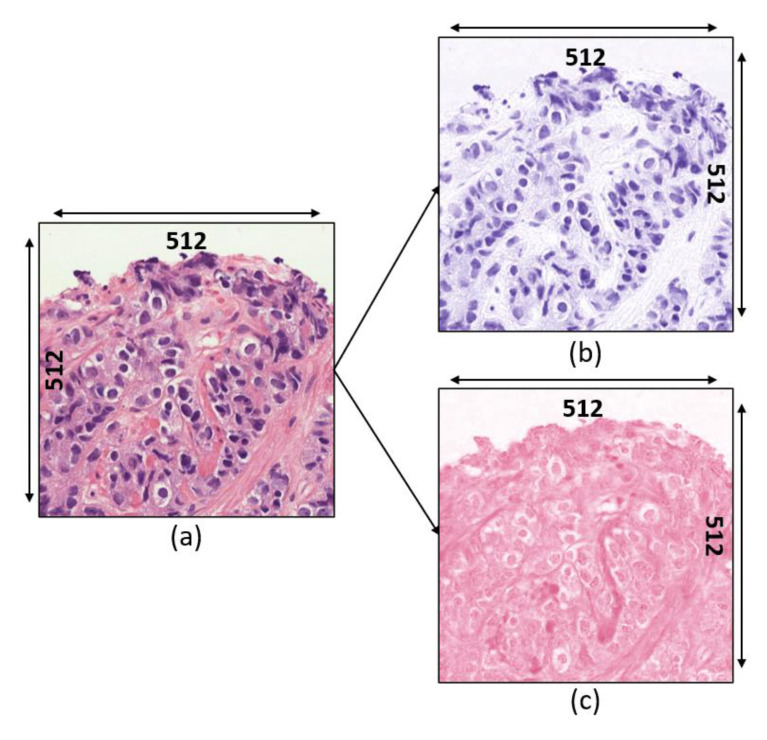
Stain deconvolution. (**a**) Normalized image. (**b**) Hematoxylin channel. (**c**) Eosin channel.

On the other hand, stain deconvolution [24] was applied to transform the RGB color image into stain color spaces (i.e., H&E). Examples of separated stain images are shown in Figure 4. All color values on the normalized image $I_{N}$ are converted to their corresponding optical density (OD) values and the computation of OD for each (Red, Green, and Blue) channel can be expressed as follows:

(5) $$I_{O}=255\;OD=-\log\left(\frac{I_{N}}{I_{O}}\right)$$

where $I_{O}$ is the background brightfield (i.e., the intensity of light entering the image).

The stain matrix $M_{H,E}=\left[\begin{array}{ccc} Red & Green & Blue\\ 0.587 & 0.754 & 0.294\\ 0.136 & 0.833 & 0.536 \end{array}\right]$ was estimated using the Qupath open-source software based on the reference image used for stain normalization. Here, $M_{H}$ is the hematoxylin stain matrix [0.587 0.754 0.294] and $M_{E}$ is the Eosin stain matrix [0.136 0.833 0.536]. The normalized image is transformed into an optical density space to determine the concentration of the individual stain in RGB channels. Furthermore, estimated stain vector channels were recombined to obtain the stained images. The computation process for determining the stain concentration and recombining the stain vector channels can be expressed as:

(6) $$Stain\;Concentration_{H,E}=OD/M_{H,E}$$

(7) $$Stain\;Image_{H}=I_{O}\times e^{(Stain\;Concentration_{H})\times \left(-M_{H}\right)}\;$$

(8) $$Stain\;Image_{E}=I_{O}\times e^{(Stain\;Concentration_{E})\times \left(-M_{E}\right)}.$$

#### 3.2.2. Nuclear Segmentation of Cancer Cells

**Figure 5:**
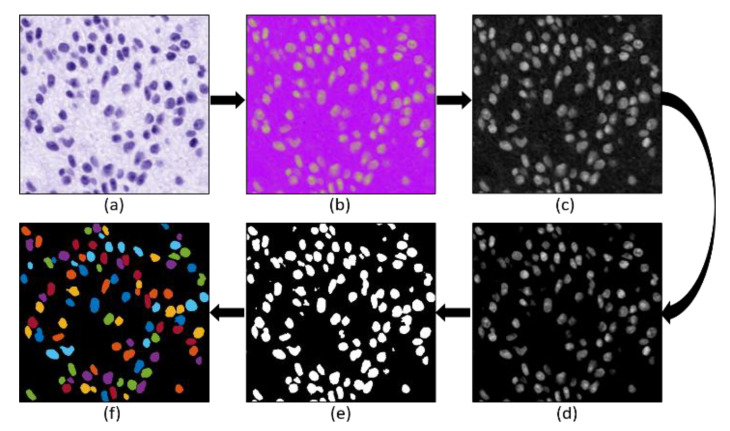
The complete process for nuclear segmentation of cancer cells. (**a**) Hematoxylin channel extracted after performing stain deconvolution. (**b**) HSI color space converted from (**a**). (**c**) Saturation channel extracted from (**b**). (**d**) Contrast adjusted image extracted from (**c**). (**e**) Binary image after applying global thresholding on (**d**). (**f**) Nuclei segmentation after applying the watershed algorithm on (**e**). Some small objects and artifacts were removed before and after applying the watershed algorithm.

To perform cell nuclear segmentation, image preprocessing was carried out as discussed in the previous section. The hematoxylin-stained image separated from the normalized image was converted to HSI (i.e., Hue—H, Saturation—S, and Intensity—I) color space. Furthermore, the image of the S-channel (8-bit/pixel) was selected for the segmentation purpose because the cell nucleus is more apparent. Next, the contrast adjustment (i.e., specifying the contrast limit) was performed to remove the inconstancy intensity from the background. Then, the global threshold method was applied to the saturation-adjusted image to convert it into a pure binary image (1-bit/pixel). Finally, the marker-controlled watershed algorithm was applied to separate the overlapping nuclei [18,25,26,27,28,29]. After separating the touching nuclei, some artifacts and objects were rejected (considered as noise), and morphological operations (i.e., closing and opening) were applied to remove the peripheral brightness and smooth the membrane boundary of the cell nucleus. Figure 5 shows the complete process for nuclear segmentation of cancer cells.

#### 3.2.3. Cluster Analysis

**Figure 6:**
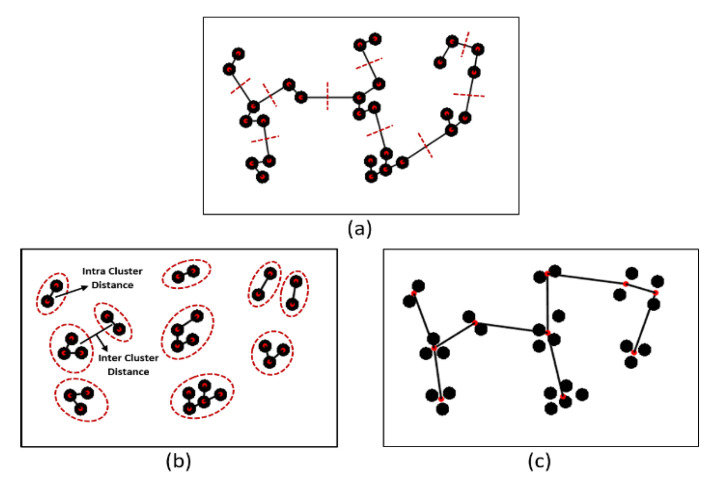
Examples of MST cluster analysis. (**a**) An MST is based on the minimum distances between vertex coordinates. The red dashed lines indicate the removal of inconsistent edges. (**b**) An intra-cluster MST was obtained after removal of the nine longest edges from (**a**); the red circles indicate inter- and intra-cluster similarity. (**c**) The inter-cluster MST was obtained from (**b**).

This study performed an intra- and inter-cluster analysis using an MST algorithm that identifies inconsistent edges between the clusters. This is a graph-based method that creates a network by connecting *m* points in *n* dimensions. Here, we used an MST for cluster analysis of cell nuclei in the histological section. In the MST, the sum of the edge weights is less than or equal to the sum of the edge weights of every other spanning tree [15,30,31]. An MST sub-graph traverses all vertices of the full graph in a cycle-free manner, yielding the minimum sum of weights of all included edges, as shown in Figure 6.

The MST usefully identifies nuclear clusters; the centroids connecting all nuclei create a graph that can be used to extract different kinds of features. Each center point of the cell nucleus, called a “vertex”, is connected to at least one other through a line segment, which is called an “edge”. We used the Euclidean minimum distance algorithm to measure the length between the two vertices its joins and construct the MST graph. The edges (distances) are sorted in ascending order and then listed. The edges pass through all vertices; if an edge connects a vertex coordinate that was not linked previously, that edge will be included in the tree [32,33]. To create separate vertices (nuclei), we used a maximum distance/weight threshold of 10 pixels. Any longer edge distance was considered inconsistent and thus removed, as shown in Figure 6a. If there are *K* vertices, the complete tree has (*K* − 1) edges. As shown in Figure 6b, the graph contains 10 groups of clusters formed by cutting links longer than a threshold value.

Next, we performed inter- and intra-cluster analyses; we computed the distances between objects in different clusters and objects in the same clusters. Cluster analysis does not require a specific algorithm; several methods are explored on a case-by-case basis to obtain the desired output. It is important to efficiently locate the clusters. Inter- and intra-cluster similarity are vital for clustering, as shown in Figure 6b,c, respectively. Cluster analysis identifies nuclear patterns and community structure in the histological sections and identifies similar groups in datasets. Data are clustered based on their similarity [34,35]. The Euclidean distance measure used to compute the distance between two data points can be expressed as:

(9) $$dist_{e}\left(x_{1},x_{2}\right)=\sqrt{\sum^{\;}{\left(x_{1}-x_{2}\right)}^{2}}$$

(10) $$dist_{inter}\left(C_{1},C_{2}\right)=\left[\left\{\left(\frac{1}{\left|C_{1}\right|}\underset{x_{1}\in c_{1}}{\sum }x_{1}\right),\left(\frac{1}{\left|C_{2}\right|}\underset{x_{2}\in c_{2}}{\sum }x_{2}\right)\right\}dist_{e}\left(x_{1},x_{2}\right)\right]$$

(11) $$dist_{intra}\left(C_{1}\right)=\left[\left(\frac{1}{\left|C_{1}\right|}\underset{x_{1},x_{2}\in c_{1}}{\sum }x_{1},x_{2}\right)dist_{e}\left(x_{1},x_{2}\right)\right]$$

where $dist_{e}\left(x_{1},x_{2}\right)$ is the Euclidean distance, $x_{1},x_{2}$ are the centroid points, and $dist_{inter}\left(C_{1},C_{2}\right)$ and $dist_{intra}\left(C_{1}\right)$ are the inter- and intra-cluster distances, respectively.

**Figure 7:**
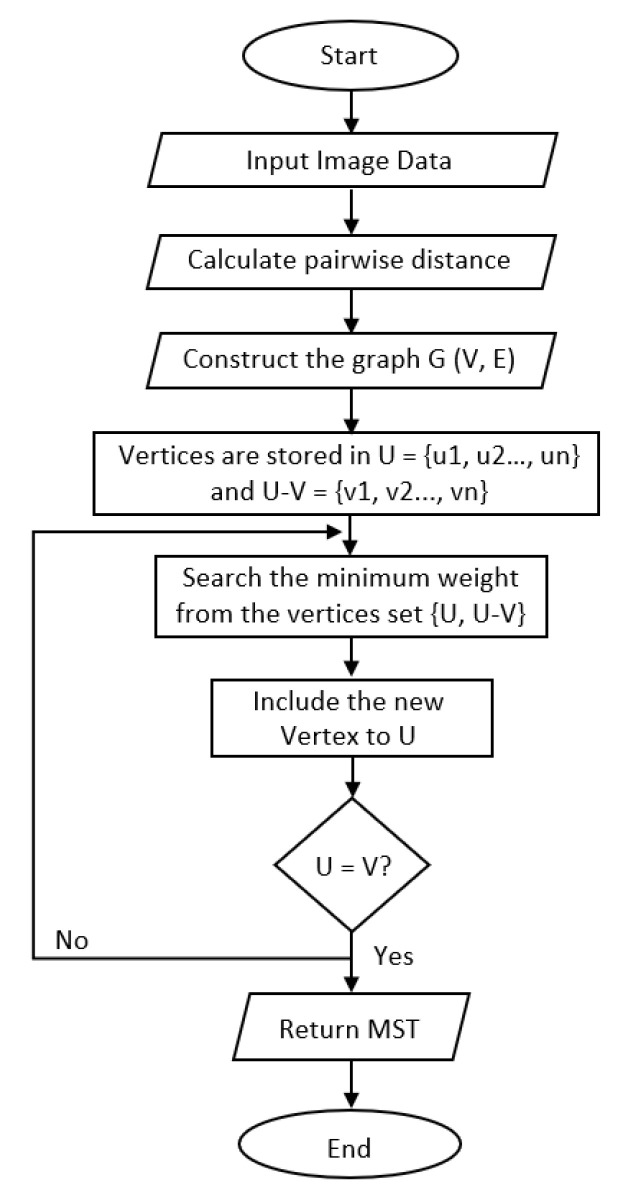
Flow chart of MST construction.

Figure 7 shows the flowchart of MST construction and the detailed algorithm is composed of the following steps:

1. Create an adjacent grid matrix using the input image.
2. Calculate the total grid numbers in the rows and columns.
3. Generate a graph from an adjacent matrix, which must contain the minimum and maximum weights of all vertices.
4. Create an *MST-set* to track all vertices.
5. Find a minimum weight for all vertices in the input graph.
6. Assign that weight to the first vertex.
7. As the *MST-set* does not include all vertices:
  - Select a vertex *u* not present in the *MST-set* that has the minimum weight;
  - Add *u* to the *MST-set*;
  - Update the minimum weights of all vertices adjacent to *u* by iterating through all adjacent vertices. For every adjacent vertex *v*, if the weight of edge *u*-*v* is less than the previous key value of *v*, update that minimum weight;
8. Iterate step 7 until the MST is complete.

#### 3.2.4. Feature Extraction and Selection

We now discuss morphological and distance-based features extracted from histological sections. Both morphological and distance-based features were used for supervised and unsupervised classification using traditional and modern AI techniques. The features were extracted as numbers based on the area and distance. A total of 26 features were extracted, which include the total intra-cluster total MST distance, total intra-cluster nucleus to nucleus maximum distance, inter-cluster centroid to centroid total distance, inter-cluster total MST distance, number of clusters, total intra-cluster maximum MST distance, average intra-cluster nucleus to nucleus minimum distance, average intra-cluster nucleus to nucleus maximum distance, average intra-cluster maximum MST distance, average cluster area, total intra-cluster nucleus to nucleus total distance, total intra-cluster minimum MST distance, total intra-cluster nucleus to nucleus minimum distance, inter-cluster maximum MST distance, average intra-cluster total MST distance, average intra-cluster minimum MST distance, total cluster area, inter-cluster average MST distance, average intra-cluster nucleus to nucleus average distance, inter-cluster centroid to centroid average distance, minimum area of a cluster, average intra-cluster nucleus to nucleus total distance, inter-cluster centroid to centroid minimum distance, inter-cluster centroid to centroid maximum distance, maximum area of a cluster, and inter-cluster minimum MST distance.

We checked the significance of each feature; this is important, because irrelevant features reduce model performance and lead to overfitting. The elimination of irrelevant features reduces model complexity and makes it easier to interpret. In addition, it enables the model to train faster and improves its performance. In this study, the combination of filter (Chi-Square, ANOVA, Information Gain, and Fisher Score) [36,37,38] and wrapper (recursive feature elimination, permutation importance, and Boruta) [39,40,41] methods were used to select the significant features. Filter methods use statistical techniques to evaluate the relationship between each input variable and the target variable, whereas the wrapper method uses machine learning algorithms and tries to fit on a given dataset and selects the combination of features that gives the optimal results. However, the best 16 features out of 26 were selected based on the majority votes. Here, we have set “minimum votes = 4” as a threshold, which signifies that the features to be selected must have at least a total of 4 votes from the seven feature selection methods, and below a total of 4 votes will be rejected, as shown in Table 2.

#### 3.2.5. AI Classification

After performing feature extraction and selection, modern and traditional AI techniques were used for supervised and unsupervised classification, respectively. For supervised classification, we used ML algorithms, namely k-NN [42], RF [43], GBM [44], XGBoost [45], and LR [46]. On the other hand, for unsupervised classification, we used a traditional k-medoids clustering algorithm [47]. We subjected each model of supervised learning to five-fold cross-validation (CV); the training data were divided into five groups, and the accuracy was recorded after five trials. Similarly, the testing was also performed based on a five-fold technique. This approach is useful for assessing model performance and identifying hyperparameters that enhance accuracy and reduce error [48,49]. The histological grades were classified as binary and multiclass to compare the performance of the AI techniques.

The data were standardized across the entire dataset before classification. Every feature has a magnitude and standardized unit. Occasionally, feature scaling is required; here, we used the standard normal distribution for standard scalar scaling:

(12) $$x_{standardized}=\frac{x^{\left(i\right)}-Avg\left[x^{\left(i\right)}\right]}{\sqrt{Var\left[x^{\left(i\right)}\right]}}$$

where $x^{\left(i\right)}$ is the feature values, $Avg\left[x^{\left(i\right)}\right]$ is the mean (μ) values, and $\sqrt{Var\left[x^{\left(i\right)}\right]}$ is the standard deviations (σ) values.

**Figure 8:**
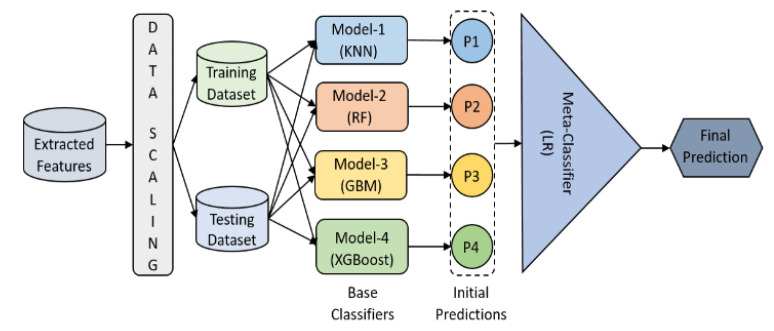
Machine learning stacking-based ensemble classification. The data were scaled before training and testing. The classification was carried out in two steps: initial and final predictions using base and meta classifiers, respectively.

We proposed an ensemble model for supervised classification, and it was designed by stacking five different machine learning algorithms. Figure 8 shows how four different classifiers get trained and tested. The initial predictions of all four base classifiers get stacked and are used as features to train and test the meta-clasifier, which makes the final prediction. The meta-classifier provides a smooth interpretation of the initial predictions made by the base classifiers. This ensemble model is developed for the higher predictive performance.

## 4. Experimental Results and Discussion

We performed qualitative and quantitative analyses to extract meaningful features and classify those using AI algorithms. Both multiclass and binary classifications were carried out to differentiate PCa grading. We subjected 900 images to preprocessing, segmentation, cluster analysis, feature extraction, and classification. The data were equally distributed among the three grades; the analyses were separate and independent. To perform supervised classification using modern AI techniques, we divided the dataset into training and testing datasets according to an 8:2 ratio. On the other hand, we used the whole dataset for unsupervised classification using a traditional AI technique. Table 3 shows the comparative analysis between supervised and unsupervised classification, and the results are based on the test dataset. Furthermore, the test and whole datasets were separated into five-split while testing our ensemble supervised model and performing k-medoids unsupervised classification for determining model generalizability. We used MATLAB (ver. R2020b; MathWorks, Natick, MA, USA) and Python programming language for stain normalization, nuclei segmentation, MST-based cluster analysis, feature extraction, and AI-based classification. The equations used for computing the performance metrics/statistical parameters can be expressed as:

(13) $$\mathrm{Accuracy}=\frac{\left(TP+TN\right)}{\left(TP+TN+FP+FN\right)}\times 100$$

(14) $$\mathrm{Precision}=\frac{\left(TP\right)}{\left(TP+FP\right)}\times 100$$

(15) $$\mathrm{Recall}=\frac{\left(TP\right)}{\left(TP+FN\right)}\times 100$$

(16) $$F1-\mathrm{score}=\frac{2\times \left(Precision\times Recall\right)}{\left(Precision+Recall\right)}$$

where TP is a true positive (correct classification of positive samples), TN is a true negative (correct classification of negative samples), FP is a false positive (incorrect classification of positive samples), and FN is a false negative (incorrect classification of negative samples).

**Figure 9:**
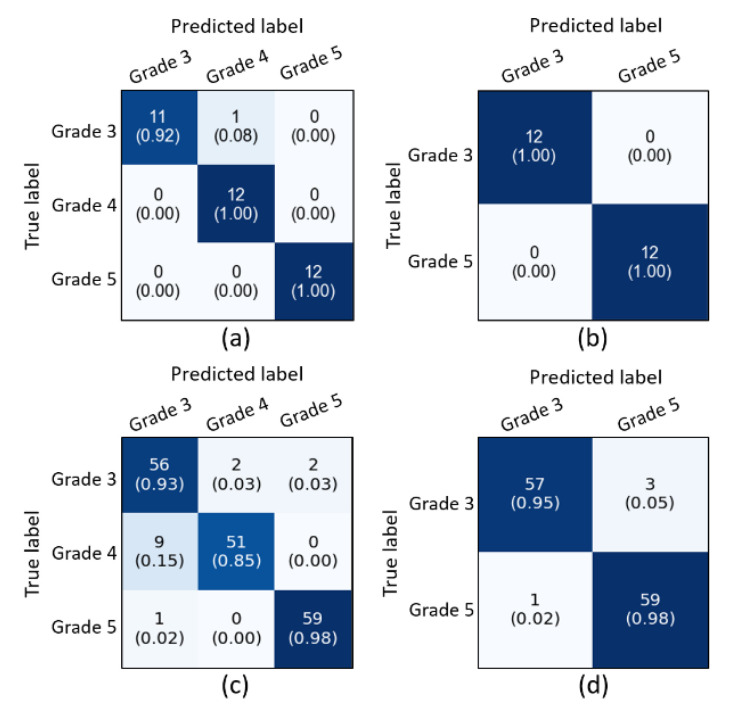
Confusion matrices of the supervised and unsupervised classification using test and whole datasets, respectively. (**a**,**b**) Confusion matrices of multiclass and binary classification using supervised ensemble technique based upon the test split 1 and 2 in Table 3A, respectively. (**c**,**d**) Confusion matrices of multiclass and binary classification using an unsupervised technique based upon the data split 2, respectively.

**Figure 10:**
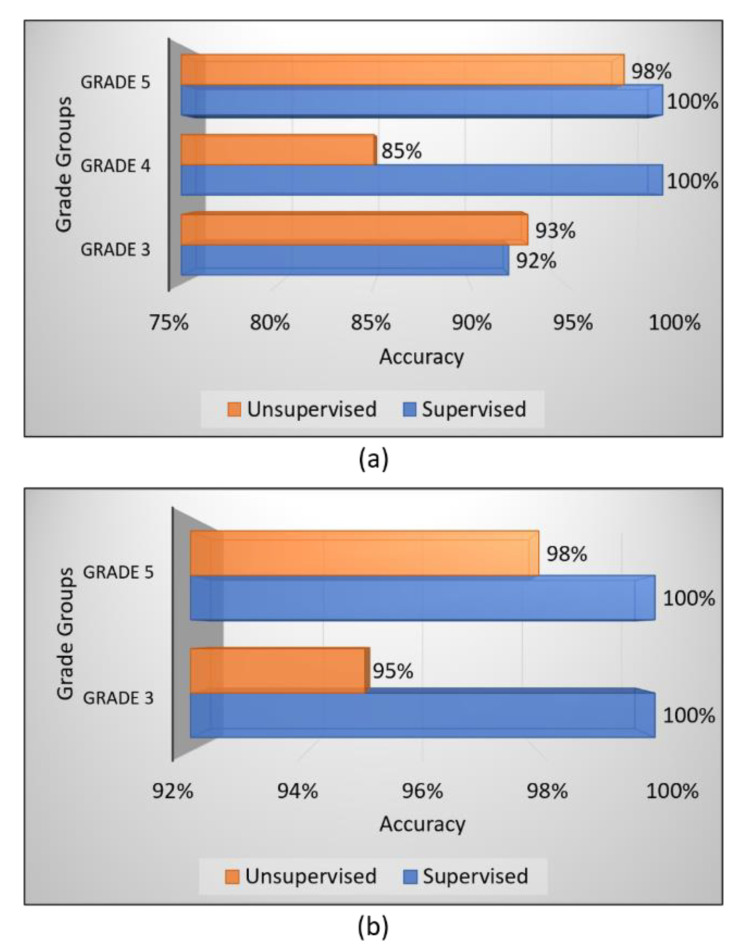
Bar charts of the accuracy scores of unsupervised and supervised classifications. (**a**) Multiclass classification. (**b**) Binary classification. The performance of each PCa grade was obtained from the confusion matrices.

From the obtained results, we have analyzed that the supervised ensemble classification using modern AI techniques outperformed unsupervised classification using a traditional AI technique. However, both supervised and unsupervised performed well and achieved astounding results. Regarding multiclass classification using the supervised ensemble technique, the model performed the best at test split 1 and achieved an overall accuracy, precision, recall, and f1-score of 97.2%, 97.3%, 97.3%, and 97.3%, respectively. Moreover, in binary classification using the supervised technique, the model achieved amazing results of 100% for all the performance measures at test split 2. In contrast, for unsupervised multiclass classification, the k-medoids algorithm performed admirably at data split 2 and achieved an overall accuracy, precision, recall, and f1-score of 92.5%, 92.7%, 92.0%, and 92.3%, respectively. Likewise, in binary classification, the k-medoids algorithm performed exceptionally at data split 2 and achieved surprising results (i.e., accuracy: 96.7%, precision: 96.5%, recall: 96.5%, and f1-score: 97.0%). Figure 9 shows the confusion matrices generated to evaluate the performance of the supervised and unsupervised classification, and the results are based on the test dataset. We present the confusion matrices of both multiclass and binary classifications and show data that were correctly and erroneously classified during testing the ensemble model and unsupervised learning. In addition, we can observe from the confusion matrices that the high cancer grade (i.e., grade 5) was perfectly and accurately classified using supervised and unsupervised techniques. Figure 10 shows the bar graph of the accuracy score of each grade separately, and the scores were obtained from the confusion matrices, as shown in Figure 9.

**Figure 11:**
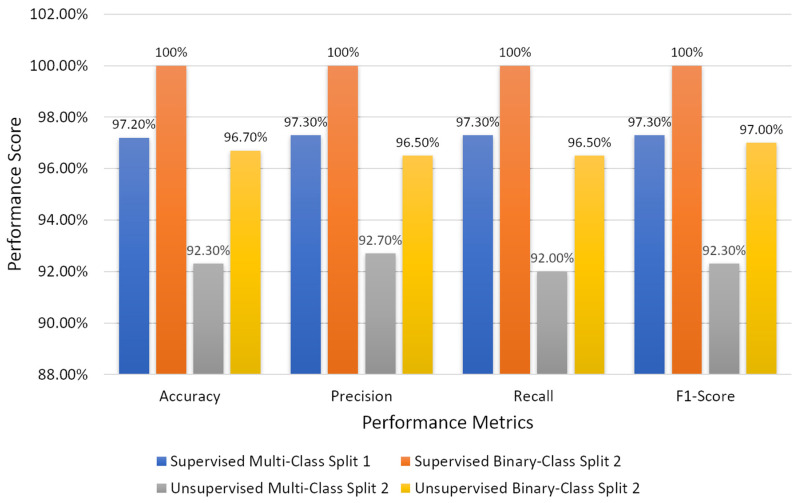
Bar chart of the overall performance scores of supervised and unsupervised classifications.

The current study was not planned using clinical data; instead, we used image data of PCa. A total of 900 microscopic biopsy samples (i.e., 300 of grade 3, 300 of grade 4, and 300 of grade 5) were selected in the present study. The data samples were distributed equally among three grade groups of PCa, and therefore, our dataset had no issue with class imbalance. For ML-based supervised ensemble classification, the dataset was separated into two parts for training (720 data samples) and testing (180 data samples) according to an 8:2 ratio. On the other hand, the whole dataset was utilized for unsupervised classification instead of divided into training and testing. In the view of feature reduction, after performing a majority voting approach using statistical and ML techniques, the 16 best features were selected based on optimum performance and 10 were rejected, as shown in Table 2. Therefore, the final selected features were used for AI classification and differentiating between the grades of PCa. Figure 11 shows the bar graph of the best performance scores of supervised and unsupervised classifications.

There are many feature selection methods, and it is quite difficult to select the best one. In addition, we need to be very concerned about the features that are being fed to the model because ML follows the rules of “garbage in” and “garbage out”. We know that irrelevant features can increase computational cost and decrease the performance of the models. However, it is challenging to identify which method is the best for our dataset, and each method has a different way to select significant features. Therefore, the majority voting approach was proposed to solve this problem.

**Figure 12:**
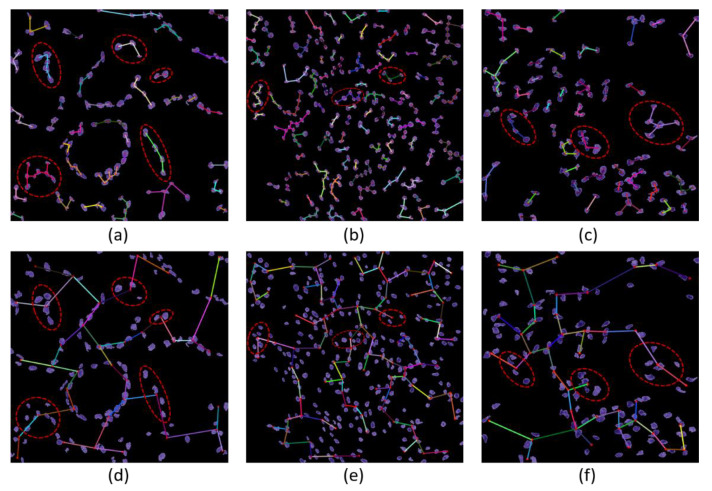
The visualization of intra- and inter-cluster MST graphs. (**a**–**c**) The intra-cluster MST of grade 3, grade 4, and grade 5, respectively. (**d**–**f**) The inter-cluster MST was generated from a, b, and c, respectively. The dotted red circle indicates the cluster of cell nuclei. Different color lines in a-c and d-f indicate intra- and inter-clusters, respectively.

The MST cluster analysis method was applied on the PCa tissue samples of dataset 1 and dataset 2, and the visualization results of intra- and inter-cluster MST are shown in Figure 12. From the following figure, we can analyze that the structure and shape of the clusters in each grade are different from each other. It is quite challenging for researchers and doctors to analyze the microscopic biopsy images of PCa and identify suitable biomarkers compared to other common cancers.

**Figure A4:**
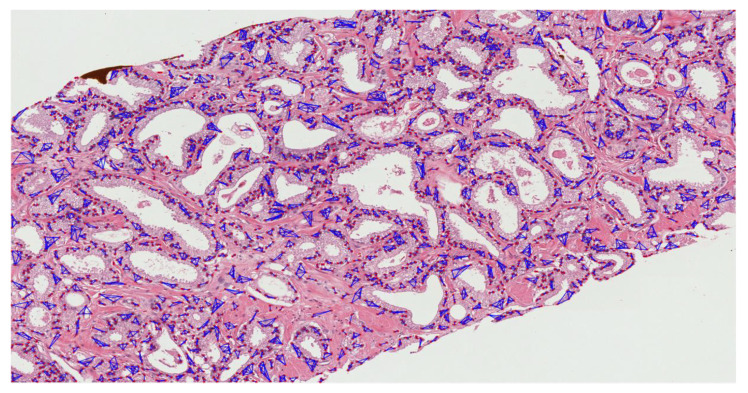
The proliferation and community structure of cell nuclei in the annotated region of grade 3.

**Figure A5:**
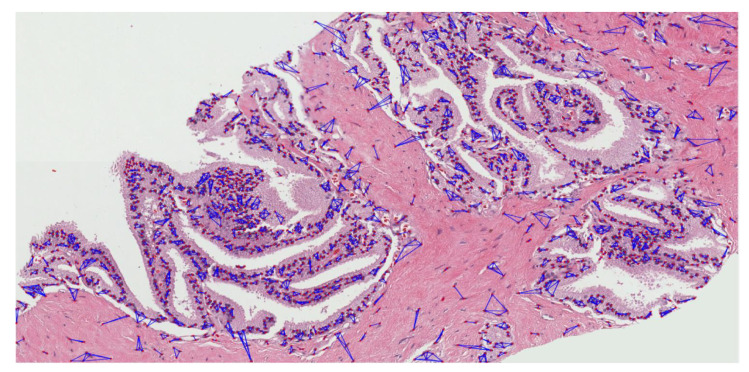
The proliferation and community structure of cell nuclei in the annotated region of grade 4.

**Figure A6:**
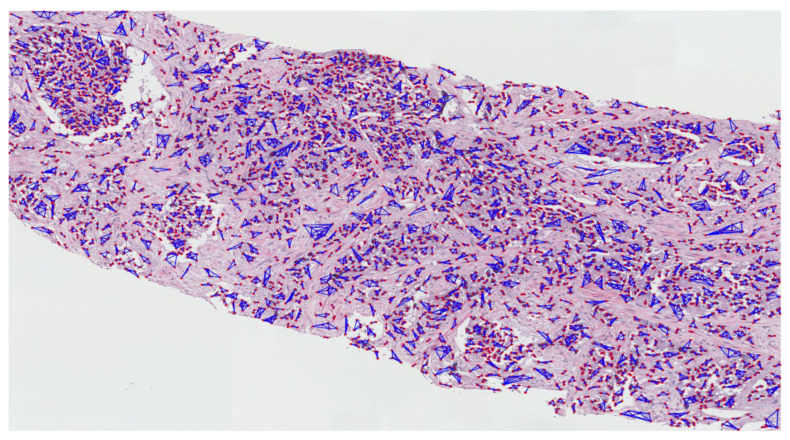
The proliferation and community structure of cell nuclei in the annotated region of grade 5.

The gold standard for the diagnosis of prostate cancer is a pathologist’s evaluation of prostate tissue. To potentially assist pathologists, DL-based cancer detection systems have been developed. Many of the state-of-the-art models are patch-based convolutional neural networks. Patch-based systems typically require detailed, pixel-level annotations for effective training. However, such annotations are seldom readily available in contrast to the clinical reports of pathologists, which contain slide-level labels. Our study sliced annotated and graded images from the pathologist, and we use an MST algorithm to perform cluster analysis and extract significant information for AI classification. The proliferation and cluster structure of cell nuclei, as shown in Appendix A, Figure A4 (Gleason pattern 3), Figure A5 (Gleason pattern 4), and Figure A6 (Gleason pattern 5), will help the pathologist to identify, classify, and grade more precisely the Gleason score assignment in the light of heterogeneity and variability.

In this era, deep learning-based algorithms are mostly used for cancer image analysis and classification. However, in this paper, we used traditional image processing algorithms to analyze PCa biopsy images and performed classification using modern and traditional AI techniques. In addition, we compared the performance of our proposed approach with the other state-of-the-art methods, as shown in Table 4.

The limitations of our study are as follows:

- The size of the image datasets was too small to perform cluster analysis and apply deep learning-based algorithms, such as graph convolution neural network (GCNN) and LSTM network, and the study could be improved by increasing the data samples.
- Cell nuclei segmentation using traditional-based algorithms is a major issue, but we can improve this problem gradually by performing cell-level analysis applying different state-of-the-art methods.
- We know that unsupervised classification is very important in the real-world environment, the classifiers used in our study performed well but did not achieve astounding results compared to supervised classification. Therefore, we can improve this problem by analyzing the feature dissimilarities between the PCa grades.

## 5. Conclusions

In the paper, we focused principally on the cluster features of nuclei in tissue images, which facilitate cancer grading. Two-dimensional tissue images stained with H&E were subjected to cluster shape and size analyses. The distribution of cell nuclei and the shape and size of the clusters have changed as the cancer grade progressed. We developed multiple methods for histopathological image analysis (i.e., stain normalization, cell nuclei segmentation, cluster analysis, feature selection, and classification). The majority voting and stacking-based ensemble techniques are proposed for feature selection and classification, respectively. All the methods were executed successfully and achieved promising results. Cell-level analysis in the field of diagnostic cytopathology is important to analyze and differentiate the clusters of cell nuclei in each cancer grade. Although we performed several types of research, many challenges remain.

In conclusion, this research contributes useful information about the proliferation and community structure of cell nuclei that exist in the histological sections of PCa. Although we used several state-of-the-art methods and achieved astounding results, in-depth research is required for the segmentation and cluster analysis of cell nuclei using other state-of-the-art algorithms. Therefore, to overcome the challenges in the field of medical image analysis, we should think beyond the borderline. In the future, we will update this research work by performing cluster-based graph convolution neural network (GCNN) classification and apply our approach to other types of cancers.

## Figures and Tables

**Table 1:** Summary of some existing papers that performed PCa analysis using histopathology images.

Author	Techniques	Classification Types	Description and Performance
Uthappa et al., 2019 [13]	CNN-based texture analysis	Multiclass (grade 2, 3, 4, and 5)	Developed a hybrid unified deep learning network to grade the PCa and achieved an accuracy of 98.0%
Khouzani et al., 2003 [14]	Handcrafted-based texture analysis	Multiclass (grade 2, 3, 4, and 5)	Calculated energy and entropy features of multiwavelet coefficients of the image and used ML classifier to classify each image to the appropriate grade. They achieved an accuracy of 97.0%
Kwak et al., 2017 [15]	CNN-based texture and nuclear architectural analysis	Binary class (benign and cancer)	The author presented a CNN approach to identify PCa. In addition, they extracted handcrafted nuclear architecture features and performed ML classification. The performance of their CNNs (0.95 AUC) was significantly better than that of other ML algorithms
Linkon et al., 2021 [16]	Different techniques related to PCa detection and histopathology image analysis have been discussed	N/A	The author discussed recent advances in CAD systems using DL for automatic detection and recognition. In addition, they discussed the current state and existing techniques as well as unique insights in PCa detection and described research findings, current limitations, and future scope for research
Wang et al., 2020 [17]	Morphological, texture, and contrastive predictive coding feature analysis	Binary class (score 3 + 3 and 3 + 4)	The author proposed a weakly supervised approach for grade classification in tissue micro-arrays using graph CNN. An accuracy of 88.6% and an AUC of 0.96 were achieved using their proposed model
Bhattacharjee et al., 2019 [18]	Morphological analysis	Binary class (benign vs. malignant, grade 3 vs. grade 4, 5, and grade 4 vs. grade 5) Multiclass (benign, grade 3, grade 4, and grade 5)	The author used histopathology images to perform morphological analysis of cell nucleus and lumen and carried out multiclass and binary classification. The best accuracy of 92.5% was achieved for binary classification (grade 4 vs. grade 5 using support vector machine classifier
Bhattacharjee et al., 2020 [19]	Handcrafted and non-handcrafted feature analysis using AI techniques	Binary class (benign vs. malignant)	The author introduced two lightweight CNN models for histopathology image classification and performed a comparative analysis with other state-of-the-art models. An accuracy of 94.0% was achieved using the proposed DL model
Nir et al., 2018 [20]	Glandular-, nuclear-, and image-based feature analysis	Binary class (benign vs. all grades) and (grade 3 vs. grade 4, 5)	Proposed some novel features based on intra- and inter-nuclei properties for classification using ML and DL algorithms and achieved the best accuracy of 91.6% for benign vs. all grades using linear discriminant analysis
Ali et al., 2013 [21]	Morphological and architectural feature analysis from cell cluster graph	Binary class (no recurrence vs. recurrence)	The author defined cells clusters as a node and constructed a novel graph called Cell Cluster Graph (CCG). In addition, they extracted global and local features from the CCG that best capture the morphology of the tumor. A randomized three-fold cross-validation was applied via support vector machine classifier and achieved an accuracy of 83.1%
Kim et al., 2021 [22]	Texture analysis using DL and ML techniques	Binary class (benign vs. malignant) and (low- vs. high-grade)	The author used DL (long short-term memory network) and ML (logistic regression, bagging tree, boosting tree, and support vector machine) techniques to classify dual-channel tissue features extracted from hematoxylin and eosin tissue images

**Table 2:** Feature selection based on majority voting. The most significant features were selected based on majority “True”. True: Selected, False: Not selected, *χ*^2^: Chi-Square Test, FS: Fisher Score, IG: Information Gain, RFE: Recursive Feature Elimination, and PI: Permutation Importance.

Features	$\chi^{2}$	FS	IG	ANOVA	RFE	PI	Boruta	Votes	Select/Reject
total intra-cluster total MST distance	True	True	True	True	True	True	True	7	Select
total intra-cluster nucleus to nucleus maximum distance	True	True	True	True	True	True	True	7	Select
inter-cluster centroid to centroid total distance	True	False	True	True	True	True	True	6	Select
inter-cluster total MST distance	True	True	True	True	True	False	True	6	Select
number of clusters	True	True	True	True	True	False	True	6	Select
total intra-cluster maximum MST distance	True	True	True	True	True	False	True	6	Select
average intra-cluster nucleus to nucleus minimum distance	False	True	True	True	True	False	True	5	Select
average intra-cluster nucleus to nucleus maximum distance	False	True	True	True	True	False	True	5	Select
average intra-cluster maximum MST distance	False	True	True	True	True	False	True	5	Select
average cluster area	True	True	False	False	True	True	True	5	Select
total intra-cluster nucleus to nucleus total distance	True	False	False	True	True	True	True	5	Select
total intra-cluster minimum MST distance	True	True	True	True	False	False	True	5	Select
total intra-cluster nucleus to nucleus minimum distance	True	True	True	True	False	False	True	5	Select
inter-cluster maximum MST distance	True	True	False	False	True	False	True	4	Select
average intra-cluster total MST distance	False	True	True	False	True	False	True	4	Select
average intra-cluster minimum MST distance	False	True	True	True	False	False	True	4	Select
total cluster area	True	False	False	False	False	True	True	3	Reject
inter-cluster average MST distance	False	False	True	True	False	False	True	3	Reject
average intra-cluster nucleus to nucleus average distance	False	False	True	True	False	False	True	3	Reject
inter-cluster centroid to centroid average distance	False	True	False	False	True	False	False	2	Reject
minimum area of cluster	True	False	False	False	True	False	False	2	Reject
average intra-cluster nucleus to nucleus total distance	True	False	False	False	False	False	True	2	Reject
inter-cluster centroid to centroid minimum distance	False	False	False	False	False	False	True	1	Reject
inter-cluster centroid to centroid maximum distance	False	False	False	False	False	False	True	1	Reject
maximum area of cluster	True	False	False	False	False	False	False	1	Reject
inter-cluster minimum MST distance	False	False	False	False	False	False	True	1	Reject

**Table 3:** Comparative analysis of the performance of supervised and unsupervised classification using test and whole datasets, respectively. A five-fold technique was used for both supervised and unsupervised classification. Split 1 and 2 from supervised and split 2 from unsupervised shows the best results marked in bold.

(A) Supervised Ensemble Classification—Modern AI Techniques				
Multiclass Classification (Grade 3 vs. Grade 4 vs. Grade 5)				
Test Split	Accuracy	Precision	Recall	F1-Score
**Split 1**	**97.2%**	**97.3%**	**97.3%**	**97.3%**
Split 2	91.7%	92.0%	91.7%	91.7%
Split 3	97.2%	97.3%	97.3%	97.3%
Split 4	94.4%	94.7%	94.7%	94.7%
Split 5	91.7%	91.7%	91.7%	91.7%
Average Split	94.4%	94.7%	94.3%	94.7%
Binary Classification (Grade 3 vs. Grade 5)				
Test Split	Accuracy	Precision	Recall	F1-Score
Split 1	91.7%	91.6	0.916	0.916
**Split 2**	**100%**	**100%**	**100%**	**100%**
Split 3	95.8%	96.2%	95.8%	95.9%
Split 4	95.8%	96.2%	95.8%	95.9%
Split 5	91.7%	92.8%	91.6%	92.2%
Average Split	95.0%	95.0%	95.0%	95.0%
**(B) K-Medoids Unsupervised Classification—Traditional AI Technique**				
Multiclass Classification (Grade 3 vs. Grade 4 vs. Grade 5)				
Data Split	Accuracy	Precision	Recall	F1-Score
Split 1	86.1%	87.0%	86.0%	86.3%
**Split 2**	**92.3%**	**92.7%**	**92.0%**	**92.3%**
Split 3	86.7%	88.3%	86.7%	87.0%
Split 4	88.3%	88.3%	88.3%	88.0%
Split 5	91.6%	91.7%	91.7%	91.7%
Average Split	88.5%	89.7%	88.3%	88.7%
Binary Classification (Grade 3 vs. Grade 5)				
Data Split	Accuracy	Precision	Recall	F1-Score
Split 1	81.7%	82.0%	81.5%	81.5%
**Split 2**	**96.7%**	**96.5%**	**96.5%**	**97.0%**
Split 3	89.2%	89.5%	89.0%	89.0%
Split 4	86.7%	87.5%	86.5%	86.5%
Split 5	93.3%	93.5%	93.5%	93.5%
Average Split	88.3%	88.5%	88.5%	88.5%

**Table 4:** Comparison with other state-of-the-art approaches. AUC: Area under the curve, DL: Deep learning, ML: Machine learning.

Authors	Methods	Classification Type		Performance
Uthappa et al., 2019 [13]	Hybrid DL	Multiclass (grade 2, 3, 4, and 5)		98.0% (Accuracy)
Khouzani et al., 2003 [14]	ML	Multiclass (grade 2, 3, 4, and 5)		97.0% (Accuracy)
Kwak et al., 2017 [15]	CNN	Binary (benign and cancer)		0.95 (AUC)
Wang et al., 2020 [16]	Graph CNN	Binary (score 3 + 3 and 3 + 4)		88.6% (Accuracy)
Bhattacharjee et al., 2019 [18]	ML	Binary	benign vs. malignant	88.7% (Accuracy)
			grade 3 vs. grade 4, 5	85.0% (Accuracy)
			grade 4 vs. grade 5	92.5% (Accuracy)
Bhattacharjee et al., 2020 [19]	DL	Binary (benign vs. malignant)		94.0% (Accuracy)
Nir et al., 2018 [20]	ML	Binary	benign vs. all grades	88.5% (Accuracy)
			grade 3 vs. grade 4, 5	73.8% (Accuracy)
Ali et al., 2013 [21]	ML	Binary (no recurrence vs. recurrence)		83.1% (Accuracy)
Kim et al., 2021 [22]	DL	Binary	benign vs. malignant	98.6% (Accuracy)
			low- vs. high-grade	93.6% (Accuracy)
Proposed	ML	Binary (Split 2)	grade 3 vs. grade 5	100% (Accuracy)
		Multiclass (Split 1)	grade 3 vs. grade 4 vs. grade 5	97.2% (Accuracy)
	K-Medoids Clustering	Binary (Split 2)	grade 3 vs. grade 5	96.7% (Accuracy)
		Multiclass (Split 2)	grade 3 vs. grade 4 vs. grade 5	92.3% (Accuracy)

## Data Availability

Dataset 1 is not available online, cannot be transferred without an internal permission procedure. It is only available on request from the corresponding author. Dataset 2 is openly available online in the Kaggle repository at https://www.kaggle.com/c/prostate-cancer-grade-assessment (accessed on 25 March 2021). Code, test data, and pre-trained models for supervised ensemble classification are available in the Github repository at https://github.com/subrata001/Prostate-Cancer-Classification-Based-On-Ensemble-Machine-Learning-Techniques (accessed on 7 September 2021).

## Appendix A

The pathology annotated WSIs used in this research to analyze the pattern and community structure of cell nuclei in grades 3, 4, and 5, shown in Figure A1, Figure A2 and Figure A3, respectively. The cluster analysis was performed successfully on histological images of PCa. For visualization of the community structure of cell nuclei, we plot the clusters in the annotated regions of grade 3, grade 4, and grade 5 in WSIs, shown in Figure A4, Figure A5 and Figure A6, respectively.
